# Ecosystem state and soil depth determine the microbial community response to warming in coastal marsh soils

**DOI:** 10.1016/j.isci.2026.116208

**Published:** 2026-06-11

**Authors:** Johanna Schwarzer, Ella Lu Logemann, Julian Mittmann-Goesele, Alexander Brodehl, Alexander Bartholomäus, Kai Jensen, Susanne Liebner, Peter Mueller

**Affiliations:** 1 GFZ Helmholtz Centre for Geosciences, Section Geomicrobiology, Potsdam, Germany; 2 Rheinland-Pfälzische Technische Universität (RPTU) in Landau, Institute for Environmental Sciences, Landau, Germany; 3 University of Münster, Institute of Landscape Ecology (ILÖK), Münster, Germany; 4 University of Hamburg, Institute of Plant Science and Microbiology, Hamburg, Germany; 5 University of Potsdam, Institute of Biochemistry and Biology, Germany

**Keywords:** environmental science, global change, microbiology, soil biology

## Abstract

Coastal marshes are important carbon sinks because soil microbial activity is often low under flooded, oxygen-deficient conditions. Global warming may alter soil microbial communities in these ecosystems and thereby affect their carbon balance, yet experimental evidence remains limited. We investigated microbial community responses to experimental warming in coastal marsh soils from two Baltic sites differing in ecosystem age and plant community composition. Marsh sods were exposed to active aboveground and soil warming in a mesocosm facility for two consecutive growing seasons. After 1.5 years, microbial community composition shifted significantly under warming, with pronounced changes in the older ecosystem but little response in the younger one. Carbon-acquiring exo-enzyme activity also showed site-specific warming effects. Our findings suggest that warming responses of soil microbial communities are not universal but depend on local factors such as successional stage, vegetation, and soil resource availability.

## Highlights

• Warming altered soil microbial community composition in a 1.5-year marsh mesocosm experiment
• Microbial responses to warming depend on local conditions
• Carbon-acquiring exo-enzyme activity also showed site-specific warming effects

## Graphical abstract

**Figure:**
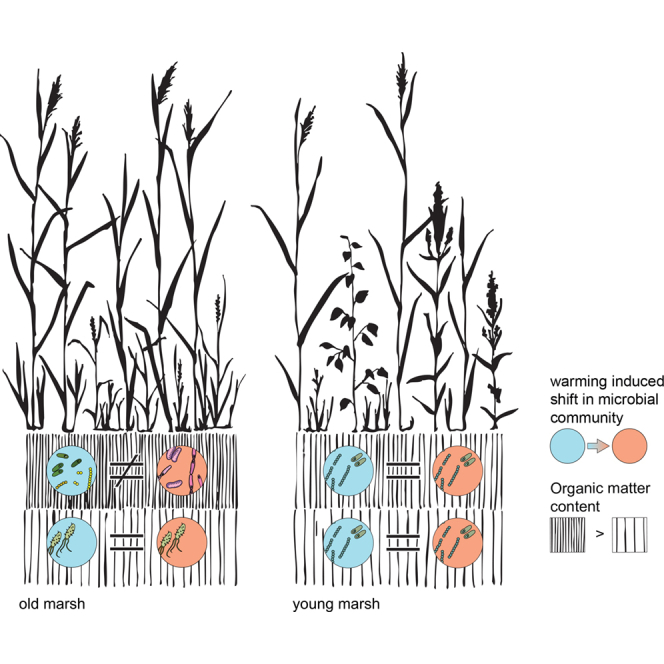

## Introduction

Global warming-driven changes in carbon fluxes between soils and the atmosphere are still poorly understood.^1^^,^^2^ Warming has the potential to mobilize large amounts of the global soil organic carbon (SOC) stock, thereby causing a release of greenhouse gases (CO_2_, CH_4_) into the atmosphere and reinforcing a positive soil-climate feedback.^3^ The conversion of SOC to greenhouse gases is driven by soil microbial respiratory processes, which have been shown to increase globally with warming over recent decades.^4^^,^^5^ At the same time, plant productivity is enhanced by warming, potentially compensating for carbon losses from soil.^2^^,^^6^ But warming-induced effects on plant productivity may level off or decrease above a certain temperature threshold.^7^ Similarly, prolonged warming potentially decreases microbial diversity and microbial SOC sequestration.^8^

Plant and soil microbial communities are key drivers of biogeochemical processes that regulate carbon fluxes.^9^ The primary source of SOC is plant-derived organic matter, and the way microbial communities process this material will ultimately determine whether SOC is stabilized or lost.^1^^,^^10^^,^^11^ Plants steer microbial functioning and composition by the quality of their litter as well as by releasing exudates and leachates that either enhance or inhibit microbial activity.^12^^,^^13^ Microbes use plant-derived organic matter as a source of carbon, energy, and nutrients. The first and rate-limiting step in the microbial decomposition of plant input to the soil is their cleavage by extracellular enzymes released by the microbial community, which makes these substrates accessible for microbial uptake.^14^ The soil microbial community modifies biotic and abiotic soil conditions and supports the ability of plants to adapt to their environment.^15^^,^^16^ Microbes make nutrients available to plants, but they also compete with plants for nutrients or are pathogenic.^17^^,^^18^ Yet, our current understanding of how climate warming influences soil microbial community functioning remains limited, partly, because the complex biotic interactions between plants and soil microbes are difficult to assess.^19^^,^^20^

Despite the potential importance of plant-microbe interactions in shaping biogeochemical dynamics in response to global warming, many studies rely on simple, plant-free soil incubation experiments to understand the effect of temperature on microbial functioning.^21^ Only few studies assess microbial warming responses in planted soils. So far, research in this field has focused on upland terrestrial ecosystems, such as forests, grasslands, and arctic tundra. Results range from significant warming-induced changes in prokaryotic community composition with warming-treatment durations of 10 years and longer,^8^^,^^22^^,^^23^^,^^24^^,^^25^ via functional gene adaptations without shifts in community structure,^26^ to studies with shorter warming-treatment durations, where warming had no detectable effect on microbial communities, particularly when compared to other environmental factors, such as soil depth, precipitation, season, and nutrient availability.^22^^,^^27^^,^^28^

Soil microbial community assembly is a deterministic process strongly governed by environmental filters such as pH, moisture, and nutrient availability.^27^^,^^28^^,^^29^^,^^30^ As succession proceeds, the influence of environmental filtering increases, leading to selective community assembly.^31^ Selective factors change dramatically with soil depth.^30^^,^^32^^,^^33^ At greater depths, experimental warming effects are often absent or less pronounced. It is argued that warming effects on microbial community composition are subordinate to environmental filters and become only apparent in the long-term and in the organic surface layers.^22^^,^^23^^,^^24^^,^^25^^,^^32^^,^^34^^,^^35^ However, the prevalence of warming effects in the soil surface may as well be a result of the experimental methods themselves, as most studies restrict temperature manipulations to aboveground heating methods such as open-top chambers or infrared heaters, or only examine surface samples.^8^

Wetlands are a key ecosystem type in the context of climate change mitigation, as they represent the largest terrestrial SOC pool.^36^^,^^37^ Their capacity to sequester and store carbon results from waterlogged soil conditions that slow down microbial activity.^38^ However, few studies to date have investigated warming effects on wetland soil microbial communities. A field study applying a strong soil-warming treatment of up to +9°C in a *Sphagnum*-dominated peat bog revealed no warming effect on microbial community composition after one year.^39^ In a follow-up study after prolonged experimental warming of 5 years, authors argue that, although they did not see a change in microbial composition, the observed increase in methane fluxes is a result of changes in microbial activity.^40^ Another field study with open top chambers for passive experimental warming (up to +0.6°C) conducted in a coastal salt marsh also found soil microbial community composition to be largely unaffected.^41^ Overall, experimental studies investigating the effects of warming on soil microbial community composition and functioning in wetland ecosystems are scarce, limiting our ability to predict the vulnerability of the vast SOC stocks stored in global wetlands such as peatlands and coastal marshes to climate change.

In the present study, we analyzed soil microbial community composition and activity in an ecosystem-transplant approach with vegetated marsh sods from two Baltic coastal marsh ecosystems differing in age and plant community composition. The marsh sods were transferred to a mesocosm facility with active above- and belowground heating of +3°C and +6°C maintained for two subsequent growing seasons. Microbial activity was assessed via exoenzyme activity (EEA) assays, while potential changes in community composition were evaluated using 16S rRNA gene sequencing.

We hypothesize that: (1) Microbial community composition is primarily shaped by soil depth, organic matter content, and plant community composition. (2) Warming induces shifts in the soil microbial community composition, especially in the organic-rich surface soil. As a consequence, we hypothesize (3) that microbial community responses to warming differ between the two ecosystems, characterized by differences in plant community composition, age, and organic matter content.

## Results

### Microbial community composition

We used 16S rRNA gene amplicon sequencing for microbial community composition analysis to assess the effect of warming treatments (ambient, +3°C, +6°C) in marsh sods of two origins (old site Denmark; young site Sweden), at three different soil depths (0–5 cm, 5–10 cm, and 10–20 cm). The ambient treatment reflects the temperature conditions at the experimental site in Hamburg, Germany. The +3°C and +6°C treatments were applied relative to the ambient baseline, where the +3°C treatment captures a realistic warming scenario, and the +6°C treatment captures the extreme end of future projections, including prolonged heatwaves.^42^ The two sites belong to the same ecosystem type and were both dominated by *Phragmites australis*, but they vary in ecosystem age and have different histories that shaped their soil development and their microbial legacy. We sampled three soil depths because microbial communities exhibit strong vertical stratification^30^ and potentially show variable warming responses in relation to soil depth.

**Figure 1:**
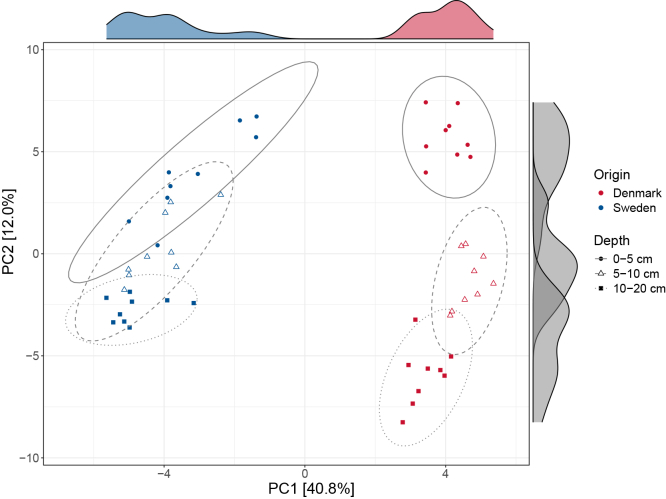
Principal component analysis (PCA) of center log-ratio transformed 16S rDNA community composition across all samples Marsh-sod origin (red: Denmark, blue: Sweden); Sampling depth (circles and straight-lined hull: 0–5 cm, triangles and dashed hull: 5–10 cm, squares and dotted hull: 10–20 cm). Side density distribution along the x axis represents the marsh-sod origin, side density distribution along the y axis sampling depth.

Prokaryotic community composition was clustered significantly by marsh-sod origin and sampling depth. Marsh-sod origin explained 40% of the variance in community composition along the first principal component (PC) (Figure 1). The sampling depth was separated along the second PC, explaining 12% of the total variance in microbial community composition. Older marsh sods from Denmark showed a stronger depth differentiation than the younger marsh sods from Sweden (Figure 1). The effects of marsh-sod origin as well as soil depth on microbial community composition were significant (*p* < 0.0001; Table S2).

**Figure 2:**
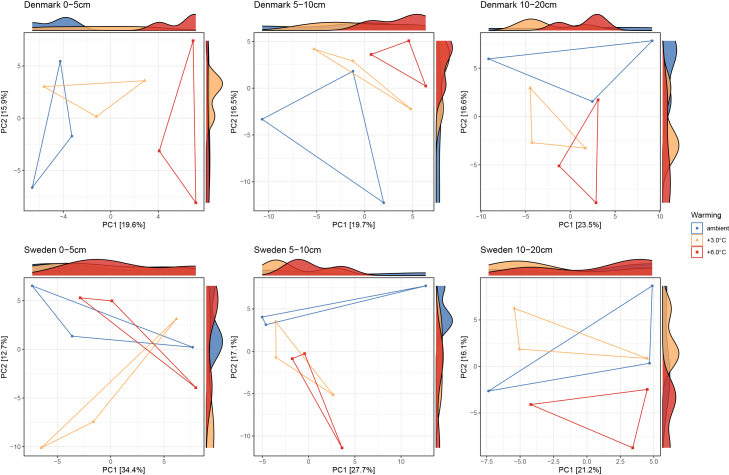
Principal component analysis (PCA) of center log-ratio transformed 16S rDNA community composition for sample subsets corresponding to the identified significant clusters (marsh-sod origin and depth; Figure 2) Marsh sods from Denmark (old ecosystem) are displayed in the top row, marsh sods from Sweden (young ecosystem) in the bottom row; sampling depths increase from left to right. Warming treatment triplicates are connected by lines (blue: ambient, orange: +3 °C, red: +6 °C).

When samples were stratified according to the main clusters, namely marsh-sod origin and soil depth (Figure 1), a warming pattern was identified (Figure 2). In the upper soil layers (0–5 cm) of the sods from the older marsh in Denmark, warming led to distinct clustering with a linear trend along the first PC. This effect was significant in the upper soil layer (PERMANOVA *p* < 0.001, Table S3) but became less apparent and ultimately non-significant with increasing soil depth.

In contrast, the microbial community composition of the sods from the younger marsh in Sweden did not exhibit a clear linear response along the primary axes, but for samples from 5 to 10 cm depth, a distinction between warming treatments is apparent (Figure 2). In surface samples from the Swedish marsh sod, samples were separated along the third and fourth PCs (Figure S1).

### Vegetation composition

Plant community composition varied strongly between marsh sods originating from older Danish and younger Swedish marshes (Figure S2). Sods from the older marsh were dominated by the late-successional grasses *Phragmites australis* and *Elymus repens*; other species, such as *Vicia cracca,* covered less than 10%. Sods from the younger marsh in Sweden had a higher plant diversity. Grasses, such as *Phragmites australis* and *Agrostis stolonifera,* were also present, as were dicots, such *as Lythrum salicaria*, *Gallium palustre,* as well as seedlings of *Alnus glutinosa* and *Betula pubescens*.

### Microbial exoenzyme activity

**Figure 3:**
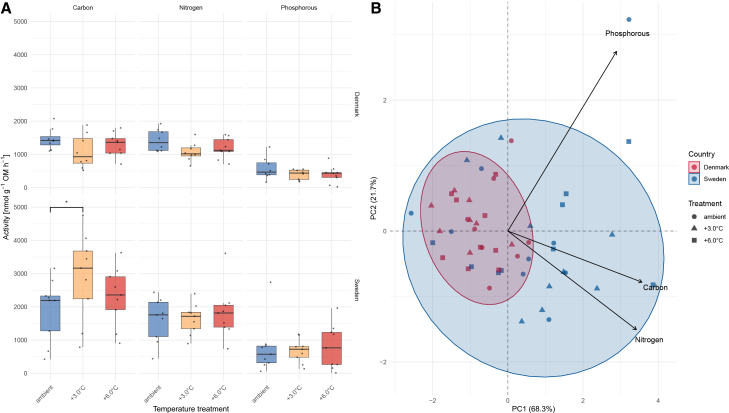
Exoenzymatic activities of carbon (β-glucosidase + cellobiosidase), nitrogen (leucine-aminopeptidase + chitinase), and phosphorus (phosphatase) acquiring enzymes, across three warming treatments (ambient, +3.0°C, +6.0°C) and two marsh-sod origins (old/Denmark, young/Sweden) (A): boxplots for individual marsh-sod origins and warming treatments, the box spans the interquartile range, and whiskers extend to 1.5 times the interquartile range, with points beyond indicating outliers. (B): PCA of carbon, phosphorus and nitrogen acquiring enzymes.

We used fluorometric EEA assays to investigate microbial activity. Sods originating from the younger Swedish marsh consistently showed higher variation in exoenzymatic activity compared to sods originating from the younger marsh in Denmark (Figure 3). Carbon (β-glucosidase + cellobiosidase, *p* < 0.001), nitrogen (leucine-aminopeptidase + chitinase, *p* < 0.01) and phosphorus (phosphatase, *p* < 0.05) acquiring enzymes were all significantly lower in marsh sods from Denmark compared to marsh sods from Sweden according to the mixed-effect ANOVA (Figure 3A). Increasing soil depth led to significantly lower activities in carbon (*p* < 0.001) and nitrogen (*p* < 0.01) acquisition. Activity in phosphorus acquisition did not differ significantly between soil depths (*p* = 0.22). While the warming treatment did not significantly affect the exoenzymatic activities (all enzymes *p* > 0.05), there was a significant interaction effect between warming treatment and marsh-sod origin regarding the activities of the carbon-acquiring enzymes (*p* < 0.01). While post-hoc pairwise comparison revealed that intermediate warming (+3.0°C) increased the microbial activity of carbon acquisition significantly in Swedish marsh sods, the effect was oppositely directed and not significant in marsh sods from Denmark (Figure 3A).

### Differential abundance of microbial ASVs

**Figure 4:**
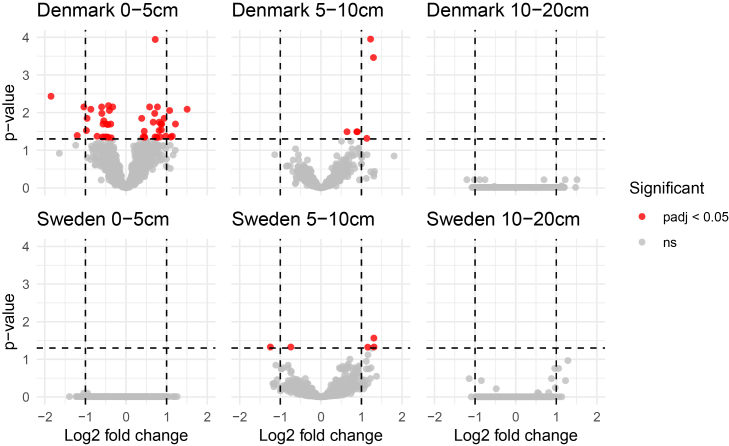
Differential abundance analysis for sample subsets corresponding to the clusters in Figure 2 Volcano plots display log2-fold changes (x axis) in ASV abundance in response to warming, with adjusted *p* values on the y axis. Each point represents an individual ASV; red points indicate statistically significant changes (Benjamini-Hochberg-adjusted *p* value <0.05) gray points are not significant (ns). Positive fold changes indicate increased abundance, and negative values indicate decreased abundance with warming.

**Figure 5:**
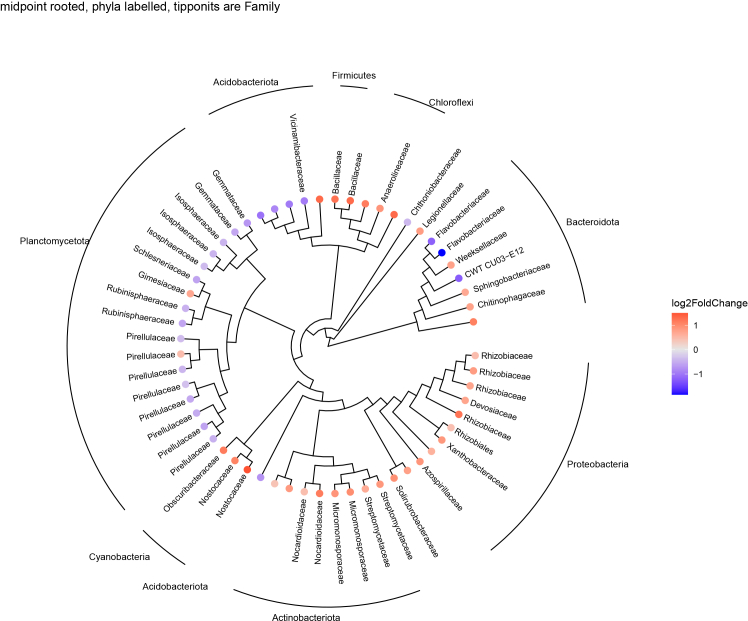
16S rRNA-based cladogram of ASVs that changed significantly in response to the warming (compare Figure 4) Tip color indicates the direction of change: red for increased abundance and blue for decreased abundance in response to warming. Tip labels are family; site labels are phylum.

The number of ASVs that significantly changed in abundance in response to warming was highest in the upper soil of the marsh sods originating from the old site, Denmark. Some responsice ASVs were also identified for soil depths of 5–10 cm in marsh sods from both Sweden and Denmark (Figure 4). The total number of ASVs that changed with warming was relatively low, making up only 1% of overall relative abundances. All identified taxa that changed with warming are displayed in the cladogram in Figure 5. About half of these ASVs increased in relative abundance with warming.

## Discussion

Our mesocosm study showed that warming can induce shifts in the microbial community composition of coastal marsh soils, even over the short period of 1.5 years. However, warming responses were strongly contingent upon environmental conditions of the system, underscoring that common broad-scale classifications, such as ecosystem type (coastal marsh) or dominance of a plant species (*Phragmites australis*) are insufficient predictors of microbial dynamics. Our results support hypothesis 1 stating that microbial community composition is strongly shaped by organic matter content, soil depth, and plant community composition (Figure 1). We can partly confirm that hypothesis 2 stating that warming induces shifts in microbial community composition, especially in the organic-rich surface soil. However, results varied for the two sites: microbial community composition in the surface soil of sods derived from Denmark was altered significantly by warming (Figure 2). In contrast, sods from Sweden did not exhibit a significant shift in microbial community composition, but a trend was apparent (Figures 2 and S1). At the same time, exoenzymatic activity, especially for carbon acquisition, showed opposing trends for the two marsh-sod origins (Figure 3). Thus, hypothesis (3), which states that microbial community responses to warming differs between the two ecosystems, is supported: The two sites exhibited divergent responses to warming, with site-specific differences appearing to drive microbial community dynamics.

### Warming effects on microbial community composition depend on ecosystem legacy and soil depth

Microbial community composition significantly changed in response to warming within only 1.5 years. While previous warming experiments in diverse ecosystems found microbial responses after prolonged warming, few studies have detected changes in microbial composition within comparable timescales.^8^^,^^22^^,^^34^^,^^39^^,^^41^ Our results highlight that microbial communities can respond rapidly to temperature increases. Considering that temperature is a fundamental environmental variable for microorganisms, it may be surprising that other studies did not observe clear temperature effects on soil microbial community composition in the short term.^19^ It has been argued that potential short-term warming effects on soil microbial communities are minor compared to the control by ecosystem type and vegetation composition, vertical stratification with soil depth, and seasonality.^39^^,^^41^ Instead, a high degree of functional redundancy within microbial communities can result in a short-term adaptation of microbial activity and metabolism in response to warming without detectable community shifts.^22^ An exception to the general absence of changes in microbial community composition under warming is the SWELTR experiment conducted in a moist lowland tropical forest of Panama, where a large shift toward thermotolerant species in response to warming (up to +8°C) has been identified after 2 years.^43^ This finding supports the notion that the sensitivity of soil microbial communities to warming is more pronounced in the tropics, where temperatures are already close to their physiological optimum.^10^^,^^19^^,^^43^

Overall, our results are consistent with previous studies showing that local environmental factors—such as site characteristics and soil depth—strongly shape microbial community composition^22^^,^^33^^,^^39^ (Figure 1). Structural controls, such as soil depth, may outweigh or mask direct warming effects on microbial assemblages. Thus, when we account for the microbial variations explained by sample origin and soil depth, as done in Figure 3, warming-induced shifts in community composition become apparent, particularly in surface soils. This suggests that temperature effects may emerge once local constraints are considered, not because warming acts independently, but because it modulates pre-existing edaphic conditions. Soil conditions are critical in shaping microbial composition and functioning through environmental filtering, and with soil depth, resource quality, oxygen availability, and moisture change drastically.^29^ In surface soils, especially of wetland systems, oxygen and nutrients are more readily available, favoring fast-growing taxa with high metabolic rates, whereas in deeper soil layers, conditions are less favorable, selecting for slower-growing microbes. Warming may lead to increased metabolic rates in the aerated nutrient rich surface soil that accelerates decomposition, while in deeper soil layers, warming effects undergo a directional shift to increased carbon sequestration in soil layers below 20 cm depth.^44^ It is likely that our study design led to a microbial warming response because of the intensity of the employed warming treatments. It has been shown that a critical predictor of warming effects on soil, at least with regard to carbon loss, is warming magnitude and duration.^44^ We increased soil temperature with active soil warming up to +6°C, whereas warming treatments in passive open-top chambers can only increase temperatures up to +2.5°C.^41^^,^^45^^,^^46^^,^^47^ But, even in studies employing active soil warming, effects on microbial community composition have not been observed within 2 years. For instance, in a temperate forest ecosystem, a shift was only detected after 20 years of continuous warming of +5 °C.^22^ In a deep peat heating experiment with warming treatments of up to +9 °C, no shift in microbial community composition was observed after 13 months of treatment.^39^ The absence of warming-related shifts in microbial community composition in active warming experiments is unlikely to be a true absence of microbial response. It is often argued that physiological adaptations outweigh compositional changes, or that the variability in microbial communities is too high to detect a consistent shift.^22^^,^^39^^,^^40^ This argument is also supported by a peatland warming experiment where a strong increase in temperature-related methane emissions for the upper peat layers was observed.^39^^,^^40^ While the main effect of our exoenzyme data cannot support this argument, the interaction effect with marsh-sod origin was significant for carbon acquisition and indicates a stronger physiological adaptation in sods from Sweden, where the shift in microbial composition was weak. The warming effect on soil microbial community composition was determined by sample origin, being pronounced in the sods from an old marsh in Denmark, but hardly detectable in the sods from a much younger marsh in Sweden. We argue that at least two non-exclusive mechanisms may explain the origin-specific response to warming: differences in ecosystem age and plant community composition. Differences in the ecosystem age between sites might explain the contrasting community response to warming. The Danish marsh site is approximately 125 years old, whereas the Swedish site is only 35 years old.^48^ This contrast in ecosystem age is particularly relevant in the context of microbial succession theory, predicting that the initial establishment of microbial communities in the early phase of ecosystem development is mainly driven by stochastic processes, meaning that the microbial community composition is formed at random. With time, the microbial community becomes increasingly structured by the local environment, to finally form a mature, strongly structured community.^31^ In support of this concept, our data demonstrates a much clearer differentiation of microbial community composition with soil depth in the sods originating from Denmark than from Sweden. Marsh sods that originated from the younger Swedish site exhibit the structure of an intermediate successional stage, where microbial communities are not yet thoroughly structured by sampling depth, but a depth pattern emerges (compare Figure 1). The higher content of organic matter of the Danish marsh sods (compare Logemann et al.^49^) indicates an older successional stage as well. Based on this framework, we interpret the different responses to warming between the two sites as follows^31^^,^^50^: In the mature, highly specialized microbial community, the warming treatment introduces a relatively strong disturbance. The more specialized community might require a stronger restructuring, which we can identify as a warming response (Figure 2). In contrast, samples from an earlier successional state, such as the Swedish marsh, harbor microbial communities that are still in the process of adapting to the environmental conditions. As a result, these communities possess higher functional redundancy and ecological flexibility, allowing them to accommodate disturbances without strong shifts in community composition. However, our result cannot confirm this theory, as the potentially greater physiological range in marsh sods from Sweden, especially for carbon acquisition, shows a warming response (Figure 3). Given the well-established bi-directional interactions between plants and microbes, the observed differences in plant diversity and community variation between marsh-sod origins likely play a key role in shaping microbial responses to warming.^41^^,^^51^^,^^52^^,^^53^ In the sods originating from Swedish marshes, we observed higher plant diversity and compositional variability with a larger share of early and mid-successional plant species (Figure S2). The higher plant diversity in the marsh sods from Sweden is likely associated with a wider range of plant traits affecting the soil microbial community, such as rooting depth and root exudation or oxygen loss.^20^ As a result, the soil microbial community may be adapted to a more diverse set of soil environmental conditions, with a wide range of physiological traits, and possibly a higher degree of functional redundancy.^50^^,^^54^ We hypothesize that this may also lead to a broader capacity of the microbial community to adapt to warming without a great shift in composition compared to the microbial communities in marsh sods originating from Denmark. However, the experimental design of this study and the obtained data are not sufficient to directly assess the relationship between plant community composition and diversity and soil microbial responses to warming.

An origin-specific warming response was also observed in exoenzymatic activities. Carbon acquisition rates tended to increase with warming for marsh sods derived from Sweden but, in contrast, tended to decrease in marsh sods from Denmark (Figure S3). This opposite warming response may be related to differences in soil organic matter content between the young and the old ecosystem.^55^ In younger and thus carbon-poorer soils, carbon availability may become increasingly limiting as temperature increases, resulting in a stimulated activity of carbon acquiring enzymes, whereas warming does not cause increasing carbon limitation in carbon-richer soils.^56^

Ecosystem age is a confounding variable, and its effect is difficult to isolate. Sods derived from Denmark and Sweden differ in climate and hydrological regimes, which influence soil development and microbial community assembly, as well as plant community composition. The older Danish marsh is dominated by two grass species, while the younger Swedish site exhibits higher plant diversity. Therefore, the legacy of plant litter and root exudates likely differs between sites. Successional stage and vegetation composition are closely linked: ecosystems in earlier successional stages typically host more generalist plant species, while late-successional systems tend to be dominated by a few well-adapted species. In addition, soil microbial communities themselves steer plant diversity, thus the described effects are bidirectional.^18^ Another important factor, not discussed, is soil physicochemical properties, which differ not only with ecosystem age but, more importantly, with sample origin. Edaphic soil parameters act as strong environmental filters that determine the composition of microbial communities and likely have contributed to the observed differences.^33^^,^^54^^,^^57^ Therefore, it is difficult to attribute the observed differences specifically to ecosystem age itself, as opposed to differences in vegetation and soil properties, or a combination thereof. Further research is needed to differentiate the individual roles in shaping microbial responses to warming.

### Taxa that increased with warming likely promote plant growth and nutrient cycling

In addition to community-level patterns, we identified microbial taxa that showed significant changes in relative abundance in response to warming, comprising approximately 1% of the total detected ASVs. Most of them were identified in marsh sods from Denmark and derived from the upper 5 cm (compare Figure 4). These findings indicate that specific prokaryotic microbes do respond to warming, even within a relatively short experimental time frame of two vegetation periods (Figure 4). Increased temperatures led to a consistent rise in *Actinobacteriota* abundance, in line with previous long-term warming studies.^23^^,^^34^^,^^58^ This response likely reflects their metabolic capacity to utilize complex carbon and nitrogen sources such as chitin and chitosan,^59^ which is in line with an increase in leaf δ^15^N isotopic signature, pointing toward the microbial mobilization of nitrogen in response to warming, supporting aboveground biomass.^49^ Microbes that increased in abundance with warming included families such as *Micromonosporaceae*, *Nocardioidaceae*, and *Streptomycetaceae*, all known for their ability to degrade complex organic compounds and contribute to nutrient cycling, particularly nitrogen and phosphorus.^60^^,^^61^^,^^62^ These functional traits may have a competitive advantage under warming, while outcompeting other taxa better adapted to nutrient-poor conditions and lower temperatures.

Similarly, microbes of the phylum Proteobacteria increased in relative abundance, which was also found in long-term warming studies.^22^^,^^58^ The family *Rhizobiacea* is closely associated with the rhizosphere, not only as a symbiotic N-fixing bacterium in legume root nodules, but also as a free-living N-fixing bacterium known to promote root growth in non-legumes.^63^^,^^64^ The only legume growing on our marsh sods was *Vicia cracca*, and it was present at low coverage, especially in those marsh sods from Denmark where the strongest microbial temperature response was observed. As the coverage of *Vicia cracca* did not respond to warming (data not shown), the increased relative abundance of *Rhizobiales* is probably linked to free-living taxa. Unlike in our study, in a host-specific study on peat mosses, *Rhizobiales* decreased in abundance under warming, indicating that host-microbe interactions may modulate warming responses differently compared to bulk soil and depending on ecosystem and location.^65^ Taxa of the family *Azospirillaceae*, also belonging to the phylum of Proteobacteria, are known to be beneficial for plants, for example, through phytohormone production that helps plants to tolerate stress or associative N-fixation, and are often closely associated with grasses.^66^^,^^67^ Nitrogen fixation is characteristic of the free-living Cyanobacteria,^68^ among which we identified three warming-responsive ASVs. The family *Obscuribacteraceae* was recently affiliated with the Cyanobacteria phylum, although they were described as anaerobic and non-phototrophs.^69^ However, this group was associated with metabolic versatility and potentially with phosphate metabolism^69^ and may, thus, also contribute to higher carbon turnover in response to rising temperatures. Two ASVs of the family *Bacillaceae* in the phylum Firmicutes increased in abundance with warming, which is consistent with other long-term studies.^34^^,^^58^ Their ability to grow rapidly when conditions are favorable, and their plant growth-promoting traits, may be advantageous under warmer conditions.^70^ Warming-responsive microbes among the phylum *Chloroflexi* are related to soil nitrogen and phosphate cycling. *Chloroflexi* can ferment complex polymers, and the family *Anaerolineae* is known as thermophylic.^71^^,^^72^ Despite mixed results in the literature, these functional capabilities may explain their higher abundance under warming in our system.^24^^,^^58^^,^^73^

Within *Bacteroidota*, responses were variable. Some taxa, particularly within *Chryseobacterium* (*Weeksellaceae*), increased under warming. This group is known for nitrogen and phosphorus cycling and for plant interaction.^74^ Others, including *Flavobacteriaceae*, decreased with warming, suggesting functional divergence within this phylum. Additional warming-responsive taxa within *Sphingobacteriales* and *Chitinophagaceae* may also contribute to enhanced nutrient turnover under elevated temperatures, as reported before.^75^^,^^76^

*Plantomycetota* generally declined in response to warming, potentially reflecting their known adaptation to cold, low-oxygen environments.^77^^,^^78^^,^^79^^,^^80^^,^^81^ This trend aligns with observations from long-term warming experiments, where similar decreases were reported.^34^^,^^76^ Although members of this phylum play important roles in nitrogen and phosphorus cycling, only two ASVs showed an increase under elevated temperatures, indicating that most *Plantomycetota* may be outcompeted in warmer conditions.^82^^,^^83^

*Acidobacteriota* were also predominantly decreasing in relative abundance with warming, aligning with previous findings that many members of this diverse phylum prefer colder conditions.^25^^,^^34^^,^^84^ Still, also within *Acidobacteria,* the temperature response was not fully consistent since one warming-responsive taxon within this group was also observed. This taxon may reflect functional heterogeneity, as members of *Acidobacteriota* were enriched in other long-term warming experiments as well.^22^^,^^24^^,^^85^

Overall, warming favored microbial taxa with traits supporting plant growth and nutrient cycling, particularly nitrogen and phosphorus mobilization. This shift may be induced directly through warming, as well as through plant-mediated selection of microbes.^12^ Conversely, declines in cold-adapted, slow-growing taxa suggest a loss of functional groups tied to more oligotrophic, low-energy systems.

### Limitations of the study

#### Context-dependent responses to warming are significant despite experimental constraints

Despite relying on a single time-point sampling, we detected responses of soil microbial communities to warming, aligning with patterns observed in long-term experiments. 16S rRNA gene sequencing captures broad shifts in communities over time and provides a robust microbial fingerprint.^86^ The detection of warming-induced shifts through 16S rRNA sequencing indicates the strength of the response, despite the temporal limitation. Capturing dynamics would require a high temporal sampling resolution and functional proxies, such as mRNA, which was beyond the scope of this study. The detection of microbial responses to warming within two growing seasons has rarely been observed, and might highlight the sensitivity of wetland ecosystems toward warming.^32^ With extended exposure to warming, the microbial response is likely to become more pronounced,^44^ but further research is needed to explore the dynamics of microbial community adaptations to warming over time. While transplanting natural marsh sods into a mesocosm facility allows for controlled conditions, the procedure inevitably disrupts hydrological connectivity and several other abiotic and biotic interactions with unkown implications for the applicatbility of our findings to field conditions. Another limitation of our study is the low degree of functional information that can be gained by 16S DNA gene sequencing. While effective for revealing community composition changes, inferences about function are indirect and based largely on reported ecological functions of known taxa. Changes in community composition do not necessarily equate to changes in ecosystem function. Thus, the functions inferred for the differentially abundant taxa should be considered putative. Similarly, EEA measures potential activity rather than *in situ* activity, as environmental controls strongly influence EEA.^87^ However, relative changes in EEA within controlled conditions are valid indicators of microbial metabolic potential.^87^ The observed warming effect in our study reflects a correlation between warming and microbial community shift, but the underlying mechanisms remain inferential. Increased temperatures can directly select microbes, or indirectly via plant-mediated effects, including altered root exudation, productivity, and plant-microbe interactions. Thus, while our data show that warming effects were evident in marsh sods from Denmark and seemed to emerge in Sweden, precise causal pathways remain uncertain. Our results highlight the importance of the local ecosystem context. While our snapshot study captures meaningful responses to environmental warming, long-term, seasonal monitoring will be critical to fully capture microbial dynamics under climate change.

## Resource availability

### Lead contact

Requests for further information and resources should be directed to and will be fulfilled by the lead contact, Johanna Schwarzer (j.schwarzer@rptu.de).

### Materials availability

This study did not generate new unique reagents.

### Data and code availability

• Sequences are uploaded to the European Nucleotide Archive (ENA): https://www.ebi.ac.uk/ena/browser/view/ERP182188.
• Original code and data are made available under the following DOI: https://zenodo.org/records/19001841.^88^
• Any additional information required to reanalyze the data reported in this paper is available from the lead contact upon request.

## Acknowledgments

We thank Gary Banta, Christoffer Boström, Johan Eklöf, Franziska Eller, Dorte Krause-Jensen, Marianna Lanari, Carmen Leiva-Dueñas, Cintia Organo Quintana and Simon Thomsen for their contributions to the conceptualization of the experimental design of the CCMMF and for collecting the marsh sods in the field. We thank Simon Thomsen, Merle Steinhagen, and Roy Rich for their technical support and implementation of the CCMMF. We thank Sarah Leonhard and Amy Senf for supporting the microbiology lab work. This research was funded through the 10.13039/501100001659DFG (Deutsche Forschungsgemeinschaft) Emmy Noether Program (502681570) and trough DFG project 451394735 (Je 264/7-1). Ella Logemann was supported by the 10.13039/501100001659DFG-funded Research Training Group 2530 (GRK2530/1-2020).

## Author contributions

J.S.: conceptualization, methodology, software, formal analysis, investigation, data curation, writing – original draft, and visualization; E.L.L.: conceptualization, methodology, formal analysis, investigation, data curation, writing – review and editing, visualization, and project administration; J.M.G.: investigation, data curation, and visualization; A.Br.: investigation; A.Ba.: software; P.M.: conceptualization, validation, resources, writing – review and editing, supervision, and funding acquisition; S.L.: validation, resources, writing – review and editing, and supervision; K.J.: resources, writing – review and editing, and supervision.

## Declaration of interests

The authors declare no competing interests.

## Declaration of generative AI and AI-assisted technologies in the writing process

During the preparation of this work, the authors used Consensus (https://consensus.app/) to find relevant academic literature. Some parts of the writing in this paper were improved with the help of ChatGPT (GPT-4, 2025), particularly for language clarity. After using these tools, the authors reviewed and edited the content as needed and take full responsibility for the content of the publication.

## STAR★Methods

### Key resources table

**Table:** 

REAGENT or RESOURCE	SOURCE	IDENTIFIER
**Biological samples**		

Baltic marsh sods derived from Sweden (for further details see Logemann et al.^49^ submitted)	58°59′12.7 ″*N* 17°36′58.4 ″E	NA
Baltic marsh sods derived from Denmark (for further details see Logemann et al.^49^ submitted)	56°44′46.9 ″*N* 10°18′14.9 ″E	NA

**Oligonucleotides**		

universal primers Uni515-F and Uni806-R	Caporaso et al.^89^	

**Critical commercial assays**		

DNeasy PowerSoil Kit	QIAGEN	NA
Qubit™ dsDNA BR Assaykit	Invitrogen	NA
HighPrep PCR – DX	MagBio Genomics Inc., Gaithersburg (Maryland), USA	NA

**Deposited data**		

Raw data	European Nucleotide Archive (ENA)	ERP182188
R code	Git via Zenodo	https://zenodo.org/records/19001841

**Software and algorithms**		

**R packages**		
phyloseq	McMurdie and Holmes^90^	version 1.52.0
microviz	Barnett et al.^91^	version 0.12.7
vegan	Oksanen et al.^92^	version 2.7–1
DESeq2	Love et al.^93^	version 1.48.2
Biostrings	Pagès et al.^94^	version 2.76.0
seqinr	Charif and Lobry^95^	version 4.2–65
msa	Bodenhofer et al.^96^	version 5.8–1
ggplot2	Wickham^97^	version 4.0.0
ggtree	Yu et al.^98^	version 3.16.3
lme4	Bates et al.^99^	version 1.1.37
emmeans	Lenths et al.^100^	version 1.11.1
tidyverse	Wickham et al.^101^	version 2.0.0
patchwork	Pedersen^102^	version 1.3.2

### Method details

#### Experimental mesocosm set-up

The Climate Change Marsh Mesocosm Facility (CCMMF) at University of Hamburg is a state-of-the-art experimental setup designed to simulate future climate warming scenarios through active, feedback-controlled warming both above and below ground (Figure S4). A detailed description of the CCMMF can be found in Logemann et al.^49^ submitted. In the CCMMF, marsh soil sods from the Baltic coast were transplanted into mesocosms in winter 2021/22. Each mesocosm consisted of six randomly assigned soil blocks from the respective sampling site, ensuring adequate mixing and representativeness. The present study is focused on a selected subset of the experiment, specifically tall-grass communities from two sites, one located in Denmark and the other in Sweden. The marsh sods were subjected to ambient, +3°C, and +6°C warming treatments. The two coastal marshes differ in ecosystem age: the Danish site emerged about 125 years ago, whereas the Swedish site is only 35 years old (under review Leiva-Dueñas et al.^48^).

#### Soil sampling and vegetation assessment

Vegetation composition was assessed in August 2023. Individual plant species cover, and bare soil was estimated in percent. After 1.5 years of active warming, soil samples were collected in November 2023. Triplicate soil cores were collected from each mesocosm using a gouge auger with a diameter of 2.5 cm. Each core was sectioned into depth increments of 0–5 cm, 5–10 cm, and 10–20 cm, which were then pooled into composite samples. Samples were stored at −20°C after sampling until further processing.

#### Microbial activity

To investigate microbial activity, we used fluorometric exoenzyme activity assays. We quantified the activities of five hydrolytic enzymes involved in microbial nutrient acquisition: carbon- (β-glucosidase, cellobiosidase), nitrogen- (chitinase, leucine aminopeptidase), and phosphorus-acquisition (phosphatase). Frozen soil samples were homogenized, and a 2 g subsample was transferred into a 50-mL Falcon tube. Soil slurries were prepared by adding 20 mL of deionized water and mixing thoroughly. Enzyme activities were measured following the standard fluorometric protocol described by.^103^ Assays were conducted in black 96-well microplates using a Multi-Detection Microplate Reader (BioTek Synergy HT, Winooski, USA). Substrates corresponding to each enzyme were added at a final concentration of 1.6 mmol/L (Table S1). Plates were incubated in the dark at 20 °C for 18–24 h. Fluorescence was measured with excitation and emission wavelengths set at 365 nm and 460 nm, respectively. Due to the high buffer capacity of the carbonate-rich soils, no additional buffering was applied before incubation. Exoenzymatic activities were normalized to the soil organic matter content.^103^ Soil organic matter was determined by loss on ignition (550°C for 2 h).

For data analysis and interpretation, exoenzyme activities were grouped accounting for the acquisition of carbon, nitrogen and phosphorus respectively. β-glucosidase and cellobiosidase were considered carbon-acquiring enzymes, chitinase and leucine aminopeptidase as nitrogen-acquiring enzymes, and phosphatase as a phosphorus-acquiring enzyme.

#### Microbial community composition

To analyze microbial community composition, we use 16S rRNA gene amplicon sequencing. Soil DNA was extracted using the DNeasy PowerSoil Kit (QIAGEN) according to the manufacturer’s instructions. Approximately 250 mg of soil per sample was used for extraction. Extracted DNA was quantified with a fluorometer (DeNovix DS-11) with the Qubit dsDNA BR Assaykit (Invitrogen), checked for size through gel electrophoresis and stored at 4°C until further processing.

For amplification of the 16S rRNA gene, samples were first diluted 1:10 with nuclease free water to dilute PCR inhibitors that are commonly found in soil samples. Amplicon libraries were prepared using the barcoded universal primers Uni515-F and Uni806-R^89^ targeting the V4 region of the prokaryotic 16S rRNA gene. The barcoded amplicons were purified with a magnetic bead clean-up step using HighPrep PCR – DX (MagBio Genomics Inc., Gaithersburg (Maryland), USA) according to the manufacturer’s protocol. The barcoded amplicons were pooled in equal quantities. Sequencing was performed by Eurofins Genomics Europe (Ebersberg, Germany) using Illumina 2x300bp paired end sequencing.

Sequencing data was demultiplexed using cutadapt version 3.4^104^ with the parameters -e 0.2 -q 15,15 -m 150 --discard-untrimmed. Amplicon sequence variants (ASVs, a proxy for phylogenetic species) were generated with trimmed reads and the DADA2 package version 1.20^105^ in R version 4.1. For this, the pooled approach with the filtering parameters maxN = 10, truncQ = 2, rm.phix = TRUE and minLen = 200 were used. Taxonomic assignment of the ASVs was done using DADA2 and the SILVA database version 138.1.^106^ ASVs that were assigned to chloroplasts, eukaryotes or mitochondria were removed.

### Quantification and statistical analysis

Statistical analysis was performed in R version 4.5.1 and RStudio.^107^^,^^108^ The 16S rRNA microbial community composition was assessed using principal component analysis (PCA) of centered log-ratio (clr)–transformed 16S rRNA gene count data^109^ with the R-packages phyloseq (v 1.52.0) and microViz (v 0.12.7).^90^^,^^91^

Community composition was also analyzed using PERMANOVA to test the influence of the factors soil depth and soil sod origin on microbial community composition with the function adonis2 of the R-package vegan (v 2.7–1).^92^ Following the same clr transformation applied for PCA (see above), community dissimilarities were quantified using the Aitchison distance, i.e., Euclidean distance on clr-transformed 16S rRNA gene count data.^109^ PERMANOVA tests were performed with 9999 permutations, using a fixed random seed (seed = 1) to ensure reproducibility. For the full dataset, we tested the effects of marsh sod origin, soil depth, and warming treatment (as continuous variable) on community composition. In addition, we repeated PERMANOVA analyses on stratified datasets to assess the isolated influence of warming treatments within each subset.

Differentially abundant ASVs in relation to the warming treatment (again modeled as continuous variable) were identified using DESeq2 (v 1.48.2).^93^^,^^110^ DESeq2 accounts for differences in sequencing depth across samples, stabilizes variability in the data, and adjusts *p*-values for multiple testing using the Benjamini–Hochberg procedure.^93^^,^^110^ The midpoint rooted Cladogram was produced by aligning the 16S rRNA sequences with the following tools: Biostrings (v 2.76.0), seqinr (v4.2-36), msa (7.3–65), ape (v 5.8–1) and plotted with ggtree (v 3.16.3).^94^^,^^95^^,^^96^^,^^111^^,^^112^

Effects of warming, marsh sod origin, depth and their interaction on enzyme activities were analyzed with mixed effect ANOVAs. Post-hoc tests were performed following significant main effects of the ANOVA. We included mesocosm replicates and sampling depth of the composite samples as a random factor into the model. Mixed effect ANOVAs were conducted using lme4 R package.^99^ Post-hoc pairwise comparisons were performed using the emmeans R package.^100^

All graphs were produced using ggplot2 (v 4.0.0) and the tidyverse (v 2.0.0) packages, with figure layouts arranged using patchwork(v 1.3.2).^97^^,^^101^^,^^102^
