# Supplementary material for: Ecosystem state and soil depth determine the microbial community response to warming in coastal marsh soils
Source: iScience. 2026 Jun 11;29(6):116208. doi: 10.1016/j.isci.2026.116208 (PMC13267566; doi:10.1016/j.isci.2026.116208)
Supplement: Document S1. Figures S1–S4 and Tables S1–S3 [file mmc1.pdf]

iScience, Volume *29*

Supplemental information

Ecosystem state and soil depth determine the microbial community response to warming in coastal marsh soils

Johanna Schwarzer, Ella Lu Logemann, Julian Mittmann-Goesele, Alexander Brodehl, Alexander Bartholomäus, Kai Jensen, Susanne Liebner, and Peter Mueller

Supplement

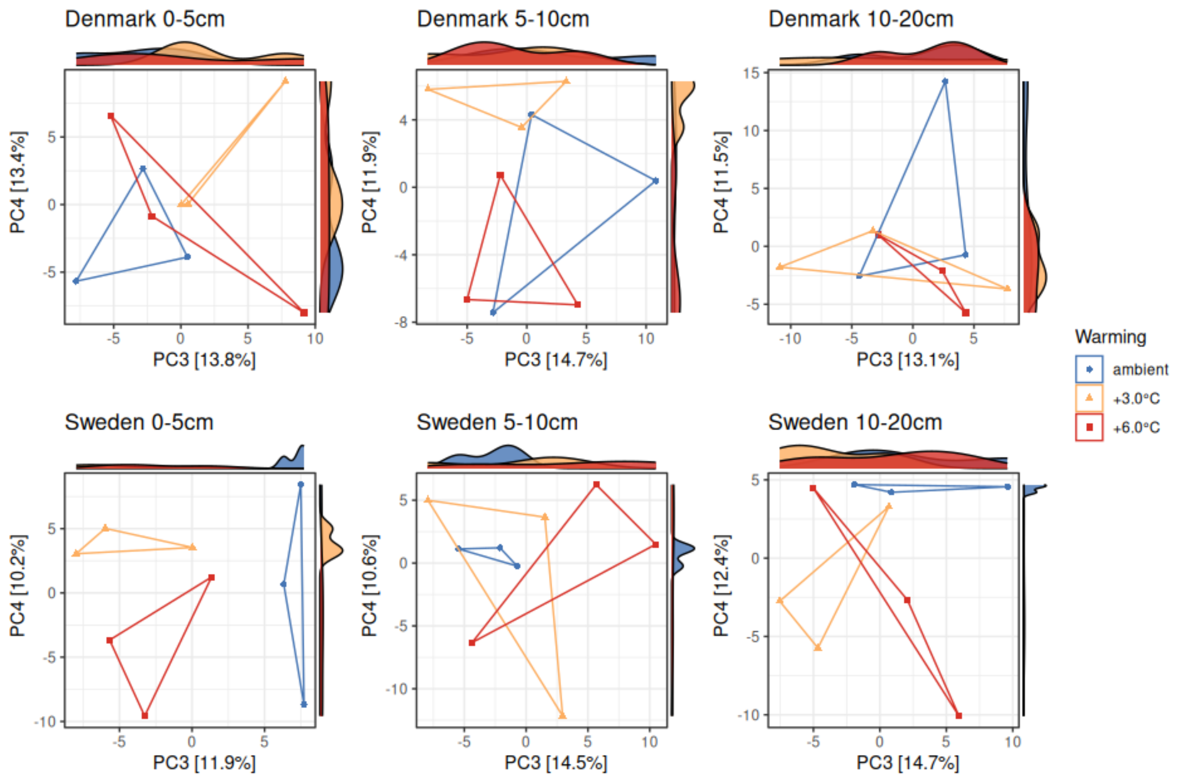

Fig. S1: Third and fourth PCs of the Principal Component Analysis (PCAs) of centre log-ratio transformed 16S rDNA community composition for sample subsets corresponding to the identified significant clusters (soil-sods and depth; Fig. 3). Marsh sods from Denmark are displayed in the top row, marsh sods from Sweden in the bottom row; sampling depths increase from left to right. Warming treatment triplicates are connected by lines (blue: ambient, orange: +3 °C, red: +6 °C)

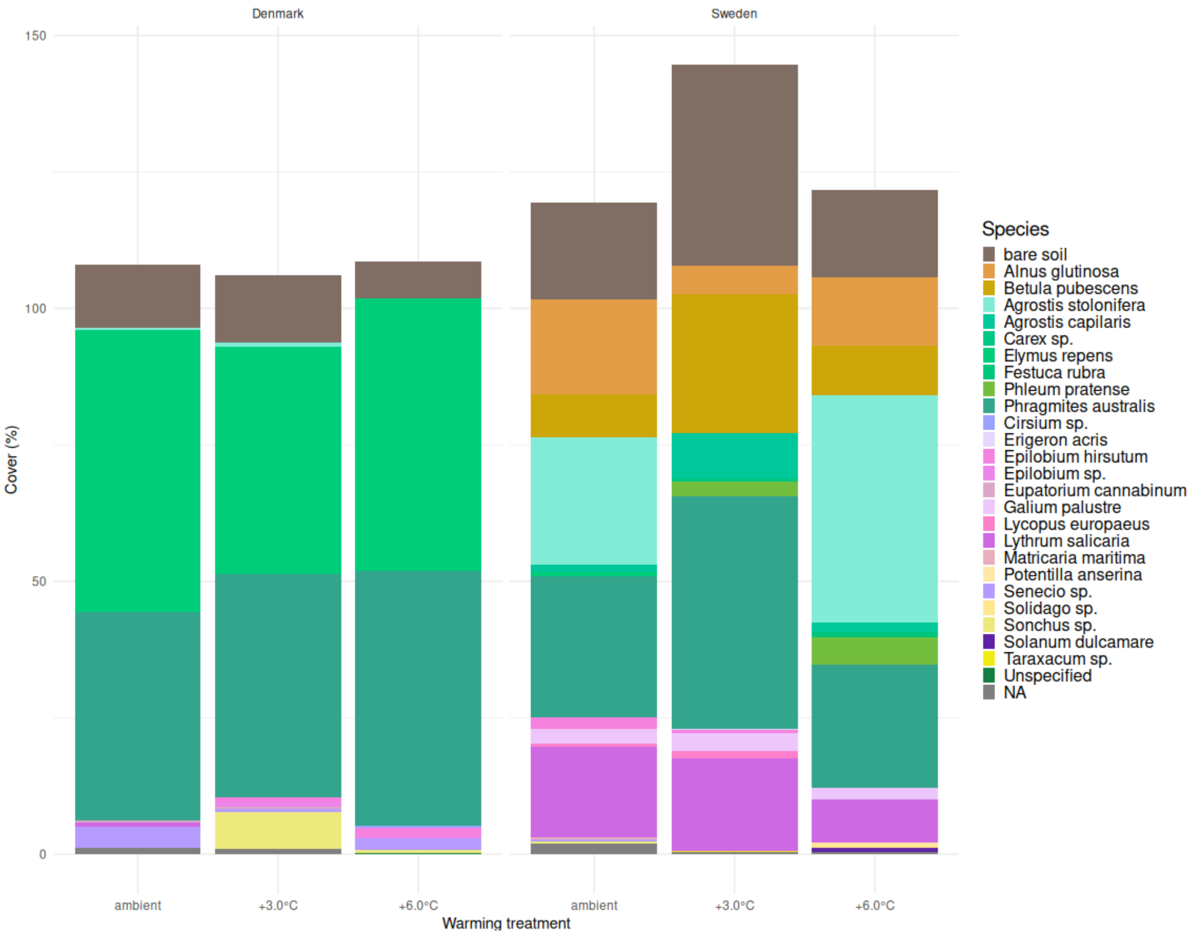

Fig. S2: Average plant community composition for marsh soil sods separated by origin (Denmark left, Sweden right) and warming treatment. Plant species are distinct by colour; grasses are in greens, trees in shades of brown and herbs in pink and yellow shades.

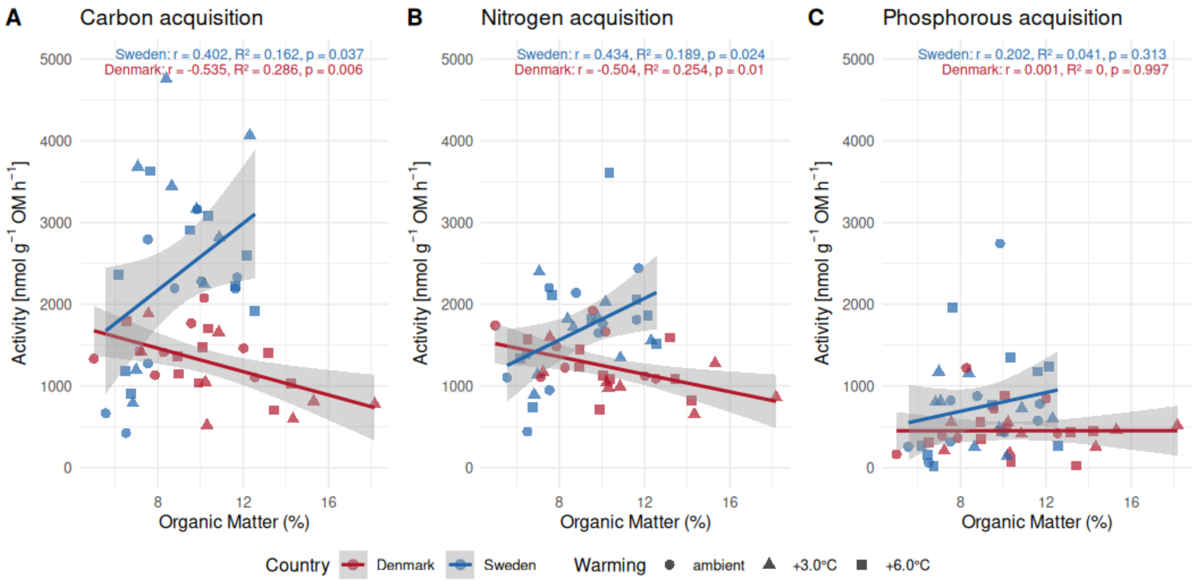

Figure S3: Exo-enzymatic activities of C, N, and P- acquiring enzymes, for all temperature treatments against soil organic matter content for the two marsh sod origins Denmark (red) and Sweden (blue).

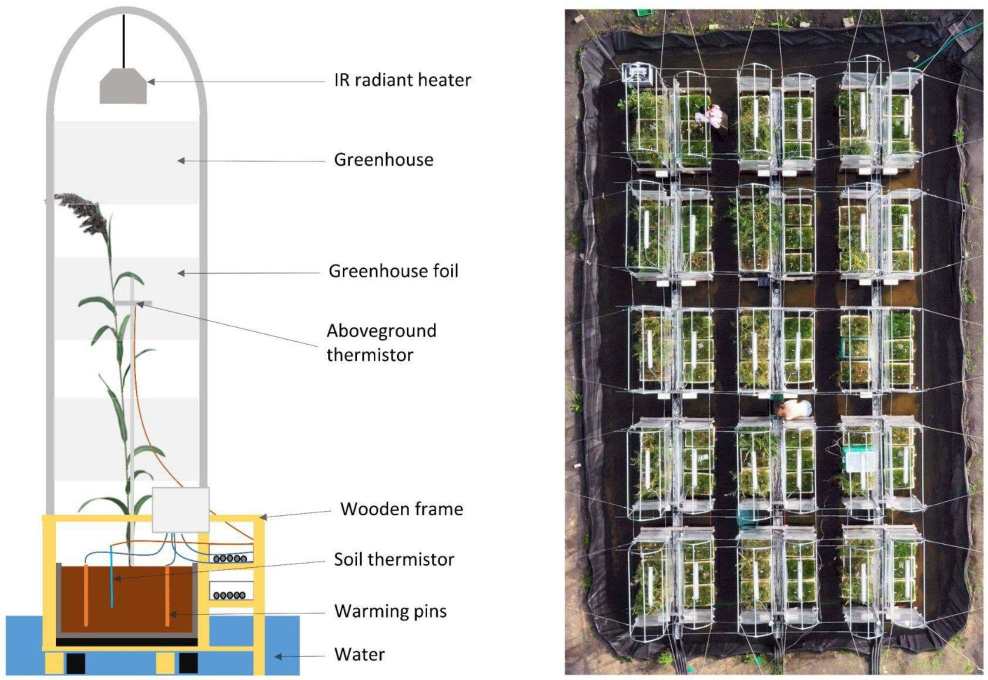

Fig. S4: Schematic overview of the CCMMF experimental setup at the University of Hamburg. Vegetated marsh sods were transplanted into mesocosms subjected to active above and below ground warming (ambient, +3°C and +6°C) using soil heating pins and infrared heaters. Mesocosms were partially submerged in a brackish water basin (6 ‰) to simulate natural water table conditions (graphic adapted from [1])

Table S1: Overview of the substrates and fluorophore standard used for exoenzyme assays

Enzyme	Substrate	Fluorophore standard (100 µmol/L)
Cellobiosidase (CEL)	4-MUF-ß-D-4-cellobioside	methylumbelliferone
β-Glucosidase (GLU)	4-MUF-ß-D-glucopyranoside	4-methylumbelliferone
Leucine-Aminopeptidase (LEU)	L-leucine-7-AMC	7-amido-4-methylcoumarin-hydrochloride
Chitinase (CHI)	4-MUF-N-acetyl-ß-D-glucosaminide	4-methylumbelliferone

Phosphatase (PHO)	4-methylumbelliferyl-phosphate	4-methylumbelliferone

Results

Table S2. Permanova result of centre log-ratio transformed 16S rDNA community composition

Factor	R2	p-value	Significance level
marsh sod origin	0.385	0.0001	***
Depth	0.126	0.0001	***
Warming	0.014	0.1501	

Table S3. Permanova results for Warming for stratified datasets of centre log-ratio transformed 16S rDNA community composition

Dataset	R2	p-value	Significance level
Denmark 0-5 cm	0.183	0.0014	**
Denmark 5-10 cm	0.148	0.0833	
Denmark 10-20 cm	0.133	0.3073	
Sweden 0-5 cm	0.117	0.4382	
Sweden 5-10 cm	0.140	0.2256	
Sweden 10-20 cm	0.135	0.3241	

1. Logemann, E.L. (2025). The Role of Plant-Soil Interactions for Carbon Cycling in Baltic and North Sea Coastal Wetlands.
